# High Prevalence of Hyperhomocysteinemia and Its Association with Target Organ Damage in Chinese Patients with Chronic Kidney Disease

**DOI:** 10.3390/nu8100645

**Published:** 2016-10-20

**Authors:** Zengchun Ye, Qunzi Zhang, Yan Li, Cheng Wang, Jun Zhang, Xinxin Ma, Hui Peng, Tanqi Lou

**Affiliations:** 1 Division of Nephrology, Department of medicine, the Third Affiliated Hospital of Sun Yat-Sen University, Guangzhou 510630, China; yzchun9@163.com (Z.Y.); kawayiqunzi@163.com (Q.Z.); wt770716@163.com (C.W.); zj_ncjx@163.com (J.Z.); maxxin_09@126.com (X.M.); elizapeng10@gmail.com (H.P.); 2 Department of Pathology, the First Affiliated Hospital of Sun Yat-Sen University, Guangzhou 510080, China; energyan@163.com

**Keywords:** chronic kidney disease, hyperhomocysteinemia

## Abstract

Hyperhomocysteinemia (HHcy) is recognized as a risk factor for cardiovascular disease. However, the prevalence of HHcy and its role in association with target organ damage in patients with chronickidney disease (CKD) are not well understood. This cross-sectional study included 1042 CKD patients who were admitted to our hospital. Patients were divided into two groups: hyperhomocysteinemia and normohomocysteinemia. Multivariable linear regression analyses were used to evaluate the association between plasma homocysteine and renal/cardiovascular parameters. The prevalence of HHcy in patients with CKD was 52.78%, and the prevalence in CKD stage 1, stage 2, stage 3, stage 4 and stage 5 patients was 10.73%, 29.22%, 58.71%, 75.23% and 83.75%, respectively. Patients with HHcy had higher incidences of renal damage, left ventricular hypertrophy, left ventricular diastolic dysfunction and abnormal carotid intima-media thickness compared with patients with normohomocysteinemia (*p* < 0.05), while multivariable linear regression analyses showed plasma homocysteine was only associated with the estimated glomerular filtration rate (eGFR). eGFR, uric acid, albumin, gender, hemoglobin and calcium×phosphate were associated with levels of plasma homocysteine in these CKD patients. The prevalence of HHcy in Chinese patients with CKD was high, and serum homocysteine levels were associated with impaired renal function in these patients.

## 1. Introduction

The rising prevalence of chronic kidney disease (CKD) has become a large public health challenge in China. One national survey showed that China had the largest number of CKD patients in the world, around 119.5 million (112.9–125.0 million) [1]. Patients with CKD have the higher risk of cardiovascular disease (CVD), and CVD is the first cause of morbidity and mortality in these patients [2]. So, exploring risk factors for CVD in patients with CKD is very important. Multiple studies have reported that decreasing the estimated glomerular filtration rate (eGFR) and increasing albuminuria are independent predictors of adverse cardiovascular outcomes in both community-based populations and in patients at high cardiovascular risk [3,4,5]. Recent studies have confirmed that traditional risk factors such as hypertension, smoking, diabetes and dyslipidemia are highly prevalent in CVD populations with CKD [6]. Apart from these, a number of nontraditional risk factors for the development of CVD have been identified based on recent studies of the pathogenesis of atherosclerosis cardiovascular events. These include high levels of plasma homocysteine, chronic inflammation, endothelial dysfunction and activation of the renin-angiotensin system [7].

Homocysteine (Hcy) is a sulfur-containing synthesized amino acid emerging on the pathway of the metabolic conversion of methionine to cysteine [8]. Total plasma levels of Hcy >15 μmol/L (Hyperhomocysteinemia, HHcy) lead to increased oxidative stress and decreased antioxidant capacities in vascular endothelial cells, which are related to increased cardiovascular risk [9]. The prevalence rates of HHcy are 5%–7% in the general population and 25% among those with vascular diseases [10,11]. However, the effectiveness of homocysteine-lowering vitamin supplementation in decreasing CVD remains controversial [12,13]. Although there is a clear association between plasma homocysteine concentration and CVD, folic acid therapy failed to prevent myocardial infarction and stroke in the majority of trials [14,15,16]. Recently, a large-scale double-blind study, China Stroke Primary Prevention Trial (CSPPT), found that the combined use of enalapril and folic acid, compared with just enalapril, significantly reduced the risk of having a stroke among adults with hypertension in China without a history of stroke or myocardial infarction [17].

Apart from playing an important role in cardiovascular and cerebrovascular diseases, HHcy is also associated with microalbuminuria and glomerular injury in general and diabetic populations [18,19]. Another study has shown that HHcy may predict the progression of renal function decline and the incidence CKD in Chinese hypertensive adults [20]. China is a country without folic acid fortification or the widespread use of folic acid supplementation and a country with a high prevalence of stroke. This necessitates further investigation into HHcy and target organ damage in China. However, data on the prevalence of HHcy in Chinese patients with CKD are lacking. We enrolled CKD patients and measured the levels of plasma Hcy, and then examined the prevalence of HHcy in these patients. We also explored the clinical parameters that were associated with HHcy.

## 2. Materials and Methods

### 2.1. Study Population

Inclusion criteria: (1) patients had to be aged 14–75 years; (2) patients who were diagnosed with CKD.

Exclusion criteria were: Undergoing treatment with corticosteroids or hormones; acute changes in the eGFR >30% intheprevious 3 months; pregnancy; history of abuse of drugs or alcohol; night work or shift-work employment; acquired immunodeficiency syndrome; cardiovascular disorders (unstable angina pectoris, heart failure, life-threatening arrhythmia and atrial fibrillation); inability to communicate and comply with all of the study requirements; on maintenance dialysis; and being in receipt of any antihypertensive drug in the previous month.

A total of 1042 CKD patients were enrolled in this cross-sectional study. In terms of causes of renal diseases: 610 patients had chronic glomerulonephritis; 136 cases had diabetic nephropathy; 296 patients had other causes of renal disease. This patient cohort was divided in two groups according to levels of plasma homocysteine: (1) normohomocysteinemia group: Patients with normal plasma homocysteine; (2) hyperhomocysteinemia group: Patients with elevated plasma homocysteine.

The study protocol was approved by the ethics committee of the Third Affiliated Hospital of Sun Yat-Sen University (Guangzhou, China). All study participants provided written informed consent to be included in the study.

### 2.2. Baseline Data Collection

Demographic characteristics and the primary cause of CKD were collected. Laboratory values for serum albumin, creatinine, cholesterol, triglycerides, low-density lipoprotein-cholesterol (LDL-C), high-density lipoprotein-cholesterol (HDL-C), uric acid, intact parathyroid hormone (iPTH) and 24 h proteinuria were collected at the first visit of the study. Plasma homocysteine was determined by ahomocysteine measurement kit (direct chemiluminescence method) using the ADVIA Centaur systems (Siemens Healthcare Diagnostics Inc., Norwood, MA, USA). Clinic blood pressure was also collected.

### 2.3. Assessment of Target Organs

#### 2.3.1. Renal Assessment

Serum concentrations of creatinine (Scr) were measured by an enzymatic method, traceable to isotope dilution mass spectrometry. We measured estimated GFR (eGFR) using the equation developed from data based on Chinese patients with CKD [21]. The CKD stages were classified using patients’ baseline eGFR according to the National Kidney Foundation/Kidney Disease Outcomes Quality Initiative NKF/KDOQI Guidelines [22].

eGFR = 175 × standardized Scr^−1.234^ × age^−0.179^ × 0.79 (if female) (1)

Scr is serum creatinine concentration in mg/dL; age is measured in years.

#### 2.3.2. Transthoracic Echocardiography Evaluation

Each patient underwent transthoracic two-dimensional echocardiography assessment by two investigators trained for this purpose before starting the study. Cardiac structure was assessed by two investigators trained for this purpose before starting the study. Left ventricular mass (LVM), systolic function, and diastolic function were assessed using two-dimensional echocardiography. Linear measurements of end-diastolic interventricular septal wall thickness (IVSd), end-diastolic left ventricular internal dimension (LVIDd), and end-diastolic posterior wall thickness (PWTd) were obtained from M-mode tracings. LVM was calculated using the following formula [23]:

LVM = {1.04 × (IVSd + LVIDd + PWTd)^3^ − LVIDd^3^} × 0.8 + 0.6 (2)

The left ventricular mass index (LVMI) was obtained by calculating the ratio of LVM to body surface area [24].

What is more, echocardiographic evaluation of left ventricle (LV) diastolic function was performed by pulsed-wave Doppler examination of mitral inflow and Doppler tissue imaging of the mitral annulus. Peak velocities of early (E) and late (A) trans-mitral flow and deceleration time (DT) were measured, and the ratio E/A was used to assess LV diastolic function.

#### 2.3.3. Carotid Ultrasonography

The assessment of Carotid intima-media thickness (cIMT) was performed as described previously [25]. Briefly, cIMT was measured using a MicroMaxx Ultrasound system paired with a 5–10 MHz Multi-frequency High-resolution Linear Transducer (SonoSite, Bothell, WA, USA). Sono-Calc IMT software was used for taking automatic measurements of cIMT. This was achieved by averaging three measurements taken on each carotid artery (anterior, lateral and posterior directions) and measuring the distance between the leading edge of the lumen–intima interface and the leading edge of the collagenous upper layer of the adventitia using high-resolution B-mode ultrasonography.

### 2.4. Definitions

Hyperhomocysteinemia was defined as plasma homocysteinemia >15 μmol/L [26].

A CKD diagnosis was made when the patient met either of the following criteria set by the Kidney Disease Outcomes Quality Initiative: (1) kidney damage is present for ≥3 months, with or without decreased eGFR, manifested by either pathologic abnormalities or makers of kidney damage (including abnormalities in blood, urine or imaging tests); (2) eGFR <60 mL/min/1.73 m^2^ for ≥3 months, irrespective of the presence or absence of kidney damage. We divided these CKD patients into five stages (1–5) according to this guideline [22].

Diabetes mellitus (DM) was defined as the need for anti-DM drugs or meeting the diagnostic criteria for DM specified by the *Chinese Guidelines for Diabetes Prevention and Treatment*: (i) symptoms of DM and casual blood glucose >11.1 mmol/L; (ii) fasting blood glucose >7.0 mmol/L [25,27].

Target organ damage (TOD) was defined as having any of the following three conditions: Firstly, impaired renal function was defined as an eGFR <60 mL/min/1.73 m^2^ [22]. Secondly, in terms of heart disease, patients with a LVMI >115 g/m^2^ (man) and >95 g/m^2^ (females) were diagnosed as having “left ventricular hypertrophy” (LVH), E/A <1 was regarded as left ventricular diastolic dysfunction [28]. Thirdly, with respect to large-vessel disease, cIMT >1 mm was regarded as an abnormal value [29].

### 2.5. Statistical Analyses

Descriptive statistics are the mean ± standard deviation (SD) for continuous variables and as the median and interquartile range for non-parametric variables. Frequency and percentage were used for categorical variables. Log transformation for proteinuria in regression analyses was undertakendue to the skewed distribution of these data.

Comparisons of continuous variables between groups were evaluated by an analysis of variance (ANOVA) or non-parametric tests. Differences among categorical variables were analyzed using the chi-squared test or the two-tailed Fisher’s exact test, as appropriate. *p* value for multiple comparisons was corrected according to the Bonferroni method (six comparisons).

Multivariable linear regression models were employed to study the association of indices of renal function (eGFR) and cardiovascular damage (LVMI, E/A ratio and abnormal cIMT) with age, gender and other variables with *p* < 0.05 explored in a univariate regression analysis.

Predictors associated with plasma homocysteine were explored by a multivariable linear regression analysis. The variables with *p* < 0.05 explored in univariate regression for this analysis were age, gender and other variables with *p* < 0.05 explored in univariate regression analysis.

All values were two-tailed and *p* < 0.05 was considered to be statistically significant. Data were analyzed using SPSS*v*20.0 (IBM, Armonk, NY, USA).

## 3. Results

### 3.1. Demographic and Clinical Characteristics of the Study Population

The mean age of the whole population was 44.34 years. Stratified by gender, the prevalence of HHcy was higher in males than in females (58.50% vs. 43.64%, *p* < 0.05). A total of 192 (18.43%) patients had diabetes mellitus, 20.92% were current smokers, and 9.98% consumed alcohol. Patients with HHcywere of older age; had a longer course; a higher prevalence of diabetes mellitus; were current smokers; had lower hemoglobin, proteinuria, cholesterol, LDL-C, HDL-C and urinary sodium excretion; and had higher clinic systolic blood pressure (clinic-SBP), serum albumin, iPTH, calcium×phosphate, uric acid, serum cystatin C, blood urea nitrogen and serum creatinine compared with subjects with normohomocysteinemia (*p* < 0.05) (Table 1).

### 3.2. Prevalence of Hyperhomocysteinemia in Different CKD Stages

**Figure 1:**
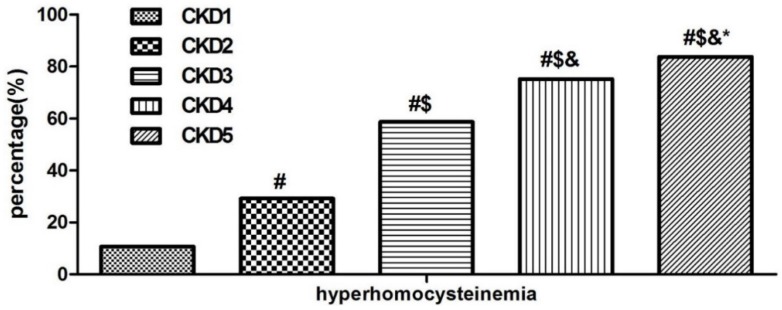
Prevalence of hyperhomocysteinemia in different CKD stages. *p*-value for multiple comparisons was corrected according to the Bonferroni method. # Comparison with CKD stage 1, *p* < 0.05; $ comparison with CKD stage 2, *p* < 0.05; & comparison with CKD stage 3, *p* < 0.05; * comparison with CKD stage 4, *p* < 0.05).

For all 1042 subjects, the prevalence of HHcy in this cohort was 52.78% (550/1042) overall, and 10.73%, 29.22%, 58.71%, 75.23% and 83.75% in CKD stage 1, stage 2, stage 3, stage 4 and stage 5 patients, respectively. Patients with poorer kidney function had a higher prevalence of HHcy than patients with better renal function (*p* < 0.05) (Figure 1).

### 3.3. Comparison of Target Organ Damage in Patients with or without Hyperhomocysteinemia

**Figure 2:**
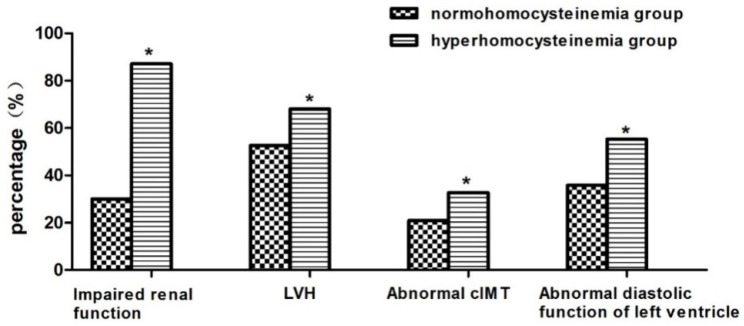
Comparison of target organ damage in patients with or without hyperhomocysteinemia. * Comparison with the normohomocysteinemia group, *p* < 0.05. cIMT, carotid intima-media thickness; LVH, left ventricular hypertrophy.

Patents with HHcy had a lower eGFR and E/A ratio, higher LVMI and higher cIMT compared with patients with normohomocysteinemia (*p* < 0.05). The incidences of renal insufficiency, left ventricularhypertrophy, left ventricular diastolic dysfunction and abnormal cIMT in patients with HHcy were higher than patients with normohomocysteinemia (*p* < 0.05) (Table 2 and Figure 2).

### 3.4. Factors Associated with Target Organ Damage in CKD Patients

Multivariate linear regression analysis showed that iPTH, hemoglobin, plasma homocysteine, age, calcium×phosphate, serum uric acid, clinic-SBP, and urinary sodium excretion were associated with eGFR. Hemoglobin, iPTH, gender, clinic-SBP and age were associated with left ventricular mass index. However, age, clinic-SBP, smoking and diabetes mellitus were associated with the E/A ratio. Age, clinic-SBP, smoking and diabetes mellitus were associated with abnormal carotid intima-media thickness (Table 3).

### 3.5. Factors Associated with Hyperhomocysteinemia and Proteinuria in CKD Patients

Multivariable linear regression analyses showed that plasma homocysteine was associated with eGFR, uric acid, albumin, gender, hemoglobin and calcium×phosphate (Table 4).

Multivariable linear regression analyses showed that proteinuria was associated with eGFR, uric acid, serum albumin, diabetes mellitus, age, clinic-SBP and triglyceride (Table 5).

## 4. Discussion

In this cross-sectional study, we investigated the prevalence of HHcy in Chinese CKD patients. Furthermore, its correlation with target organ damage and the main factors that determine HHcy were also analyzed. For all 1042 CKD subjects, the prevalence of HHcy in Chinese patients with CKD was 52.78% (550/1042) overall; the prevalence of HHcy in patients with poorer renal function was higher than patients with better renal function. Patients with HHcy had more severe target organ damage compared with normohomocysteinemia subjects, while multivariable the linear regression analyses showed that plasma homocysteine only correlated with eGFR. Age, male sex, diabetes, hypertension and hyperuricemia were associated with the presence of HHcy. All these above data suggested that HHcy is very common, especially in patients with poorer renal function. In these CKD patients, HHcy play a role in kidney damage.

HHcy has been associated with an increased risk of many diseases, such as CKD, diabetes, cardiovascular and cerebrovascular diseases [9,30,31]. In recent years, researchers have conducted a number of epidemiological studies to investigate the prevalence of HHcy in China [27,32,33]. These studies, however, were limited by a focus on the prevalence of a general population or hypertensive patients, and the prevalence of HHcy has not yet been evaluated sufficiently in the CKD population. To the best of our knowledge, this is the first study concerning the prevalence of HHcy in CKD population in China. The pooled prevalence of HHcy significantly increased from 10.73% in CKD stage 1 to 83.75% in CKD stage 5 with the deterioration of renal function. What is more, the multivariable linear regression analysis showed that plasma homocysteine was determined by eGFR, which suggested that the kidney might contribute to HHcy in these CKD patients. However, the etiology of hyperhomocysteinemia in CKD patients is still unclear.The possibilities may be due to several reasons. Firstly, decreasing kidney function can reduce the renal metabolic extraction of Hcy due to the decreasing plasma flow. A study has demonstrated that patients with renal failure had a markedly reduced clearance of Hcy from plasma [34]. In addition, extrarenal defects in homocysteine metabolism may occur in CKD patients. A study has reported that homocysteineremethylation, one of the mainly metabolized pathways in homocysteine degradation, is diminished in patients with end stage renal disease compared with healthy controls [35]. Apart from the above reasons, there is evidence to suggest that the lower dietary intake of folate and the C677T MTHFR mutation may contribute to HHcy in a general population [36]. However, further research is still needed to confirm these views in the CKD population.

Some studies have shown that HHcy plays a role in target organ damage. A meta-analysis found that a 25% lower homocysteine level (about 3 μmol/L) was associated with an 11% lower ischemic heart disease risk and a 19% lower stroke risk in healthy populations after adjustment for known cardiovascular risk factors [13]. One historical prospective study showed that annual eGFR decline was 25% higher in subjects with an elevated versus normal homocysteine level even in the general population [37], while another study demonstrated that HHcy may predict the progression of renal function decline and the incidence of CKD in Chinese hypertensive adults [20]. However, data on the role of plasma homocysteine in CKD patients remain lacking. Recently, a large cross-sectional study including 1967 patients reportedthat levels of Hcy accounted for a partial but obvious effect of renal dysfunction on the carotid total plaque area. In addition, other uremic toxins also contributed to the residual effects of renal failure on atherosclerosis [38,39]. Consistent with this study, our study found that incidences of LVH, left ventricular diastolic dysfunction and abnormal cIMT in patients with HHcy were higher than with patients with normohomocysteinemia. Patients with HHcy had more severe target organ damage than patients with normal homocysteine. However, our results, from multivariable linear regression analyses, showed that only eGFR, rather than parameters of cardiovascular risk, was associated with the levels of plasma homocysteine, which suggested that homocysteine may contribute to kidney damage in the CKD patients. The following mechanisms may be involved in renal injury due to HHcy. Increased oxidative stress and decreased antioxidant defense function, caused by a high level of Hcy, has been found to be associated with the risk of CKD [40]. In addition, it has been reported that glomerular mesangial expansion and podocyte effacement, as well as slit diaphragm injury were observed in rats even in the very early time of HHcy, which led to glomerulosclerosis and loss of renal function [41,42]. Studies have also found that Hcy caused NADPH oxidase activation, eventually leading to oxidative injury in podocytes, which was a possible pathogenic mechanism underlying the kidney toxicity of hyperhomocysteinemia [43]. Furthermore, recent research demonstrated that HHcy induced NLRP3 inflammasome formation and activation in mouse glomeruli resulting in glomerular injury or consequent sclerosis [44,45]. Recent data from the renal substudy of the CSPPT showed that CKD patients treated with enalapril-folic acid had much greater reductions in levels of serum homocysteine than those (irrespective of the presence or absence of CKD) treated with enalapril alone. Most importantly, enalapril-folic acid therapy, compared with enalapril alone, significantly slowed down the progression of kidney dysfunction among hypertensive patients with mild-to-moderate CKD [46]. Considering the significant efficacy of loweringhomocysteinewith folic acidsupplementation for reducing CKD risk, we should pay special attention to HHcy in CKD patients based on its higher prevalence and correlation with renal parameters.

The present study had strengths and limitations. Firstly, the size of the study is rather larger compared with previous studies in CKD patients, while only one center was enrolled. Secondly, all CKD patients underwent comprehensive assessments. Thirdly, all CKD patients were admitted to our hospital division, which helped to finish the assessment. Fourthly, these patients had severe proteinuria or severe renal damage, thus some CKD patients with non-severe proteinuria or non-severe renal damage might have been excluded, leading to a difference from general CKD patients. Fifthly, CKD patients who received medications, such as any antihypertensive drug in the previous month, were excluded, so drug use would not have affected analyses. However, we could not rule out the effect of Chinese medicines. Finally, we cannot set a cause–effect relationship based on this associative and cross-section study. The underlying etiology or comorbidity (e.g., hypertension, diabetes) are associated with the decline of kidney function as well. Good quality, long-term, large longitudinal trials to validate the role of plasma homocysteine in clinical practice for Chinese CKD patients are needed.

## 5. Conclusions

We have provided the first evidence ofthe prevalence of hyperhomocysteinemia and its close association with kidney damage in CKD patients in China, while we cannot set a cause–effect relationship based on this associative and cross-section study. Further prospective randomized clinical trials are needed to clarify whether lowering homocysteine treatment has a beneficial effect on attenuating the progression of renal failure in CKD patients.

## Figures and Tables

**Table 1:** Comparisonof demographic and clinical characteristics in Chinese CKD patientswith different plasma homocysteine levels.

	Total	Normohomocysteinemia	Hyperhomocysteinemia	*p* Value
	(*N* = 1042)	(*N* = 492)	(*N* = 550)	
Age (years)	44.34 ± 16.53	39.94 ± 16.40	48.27 ± 15.65	<0.001
Male: Female ratio	641:401	266:226	375:175	<0.001
Course (months)	6 (1–24)	4 (1–24)	12 (1–36)	<0.001
Diabetes mellitus (N/%)	192 (18.43%)	69 (14.02%)	123 (22.36%)	<0.001
Current smoker (N/%)	218 (20.92%)	87 (17.68%)	131 (23.82%)	<0.05
Alcohol intake (N/%)	104 (9.98%)	43 (8.74%)	61 (11.09%)	0.216
BMI (kg/m^2^)	23.24 ± 4.02	23.22 ± 4.14	23.27 ± 3.92	0.845
Clinic-SBP (mmHg)	145.38 ± 24.67	138.51 ± 22.67	151.52 ± 24.79	<0.001
Clinic-DBP (mmHg)	86.95 ± 14.46	84.88 ± 13.19	88.81 ± 15.29	<0.001
Hemoglobin (g/L)	112.77 ± 29.70	123.32 ± 27.02	103.33 ± 28.81	<0.001
Albumin (g/L)	33.93 ± 8.26	31.31 ± 9.23	36.27 ± 6.45	<0.001
Calcium×Phosphate (mg^2^/dL^2^)	38.54 ± 11.61	34.34 ± 8.98	42.26 ± 12.38	<0.001
iPTH (pg/mL)	72.05 (37.70–230.09)	42.44 (27.35–73.50)	139.56 (58.44–330.48)	<0.001
Serum fasting glucose (mmol/L)	5.24 ± 1.93	5.15 ± 1.71	5.32 ± 2.11	0.179
Cholesterol (mmol/L)	5.75 ± 2.79	6.65 ± 3.28	4.94 ± 1.95	<0.001
Triglyceride (mmol/L)	2.00 ± 1.68	2.10 ± 1.89	1.91 ± 1.46	0.066
HDL-C (mmol/L)	1.19 ± 0.44	1.30 ± 0.47	1.09 ± 0.39	<0.001
LDL-C (mmol/L)	3.70 ± 2.13	4.41 ± 2.51	3.07 ± 1.45	<0.001
Homocysteine (μmol/L)	18.14 ± 10.44	10.57 ± 2.88	24.93 ± 10.08	<0.001
Uric acid (mmol/L)	477.96 ± 152.54	419.05 ± 124.80	530.31 ± 155.92	<0.001
Proteinuria (g/24 h)	1.58 (0.52–4.03)	2.03 (0.46–5.23)	1.36 (0.55–2.90)	<0.001
Urinary sodium excretion (mmol/24 h)	123.83 ± 78.29	132.24 ± 87.15	115.31 ± 67.24	<0.05
Serum Cystatin C (mg/L)	2.69 ± 2.03	1.57 ± 1.39	3.66 ± 1.99	<0.001
Blood urea nitrogen (mmol/L)	9.87 (5.57–20.38)	5.83 (4.33–8.98)	17.20 (9.86–26.37)	<0.001
Serum creatinine (μmol/L)	158.70 (84.70–538.00)	86.00 (66.57–133.07)	428.00 (167.30–789.50)	<0.001

BMI: Body mass index; HDL-C: High-density lipoprotein cholesterol; iPTH: Intact parathyroid hormone; LDL-C: Low-density lipoprotein cholesterol; SBP: Systolic blood pressure; DBP: Diastolic blood pressure.

**Table 2:** Comparison of target organ damage in patients with or without hyperhomocysteinemia.

	Total	Normohomocysteinemia	Hyperhomocysteinemia	*p* Value
	(*N* = 1042)	(*N* = 492)	(*N* = 550)	
eGFR-MDRD (mL/min/1.73 m^2^)	38.12 (8.52–90.08)	87.68 (49.22–119.84)	11.63 (5.52–35.56)	<0.001
LVMI (g/m^2^)	124.68 ± 37.35	116.19 ± 39.92	128.99 ± 35.26	<0.001
E/A	1.11 ± 0.45	1.20 ± 0.43	1.02 ± 0.45	<0.001
cIMT (mm)	0.75 ± 0.34	0.71 ± 0.31	0.78 ± 0.35	<0.05

cIMT: Carotid intima-media thickness; eGFR: Estimated glomerular filtration rate; LVMI: Left ventricular mass index; E/A: Peak velocities of early (E) and late (A) trans-mitral flow and deceleration time; MDRD: Modification of Diet in Renal Disease.

**Table 3:** Multivariable linear regression analysis for Lg(eGFR), LVMI, E/A, cIMT.

Variables	Unstandardized Coefficients B (95% Confidence Interval)	Standardized Coefficients Beta	*p* Value
Dependent variable: Lg (eGFR by MDRD formula) (Adjusted *R*^2^ = 0.806)			
iPTH (per 1 pg/mL)	−0.001 (−0.001–0.001)	−0.297	<0.001
Hemoglobin (per 1 g/L)	0.005 (0.004–0.006)	0.285	<0.001
Homocysteine (per 1 μmol/L)	−0.01 (−0.012–0.007)	−0.204	<0.001
Age (per 1 year)	−0.011 (−0.014–0.008)	−0.217	<0.001
Calcium×Phosphate (mg^2^/dL^2^)	−0.004 (−0.006–0.002)	−0.116	<0.001
Uric acid (per 1 mmol/L)	0.000 (−0.001–0.000)	−0.111	<0.001
Clinic-SBP (per 1 mmHg)	−0.002 (−0.003–0.001)	−0.090	<0.001
Urinary sodium excretion (per 1 mmol/24 h)	0.000 (0.000–0.001)	0.048	0.038
Dependent variable: LVMI (kg/m^2^) (Adjusted *R*^2^ = 0.266)			
iPTH (per 1 pg/mL)	0.032 (0.022–0.042)	0.291	<0.001
Hemoglobin (per 1 g/L)	−0.291 (−0.414–0.167)	−0.227	<0.001
Clinic-SBP (per 1 mmHg)	0.283 (0.152–0.415)	0.188	<0.001
Gender (female = 0; male = 1)	11.872 (5.022–18.723)	0.150	0.001
Age (per 1 year)	0.233 (0.015–0.450)	0.094	0.036
Dependent variable: cIMT (mm) (Adjusted *R*^2^ = 0.295)			
Age (per 1 year)	0.009 (0.007–0.012)	0.390	<0.001
Clinic-SBP (per 1 mmHg)	0.002 (0.001–0.004)	0.152	0.001
Current smoker (no = 0, yes = 1)	0.125 (0.045–0.204)	0.140	0.002
Diabetes mellitus (no = 0, yes = 1)	0.105 (0.021–0.189)	0.120	0.015
Dependent variable: E/A (Adjusted *R*^2^ = 0.295)			
Age (per 1 year)	−0.016 (−0.018–0.014)	−0.580	<0.001
Clinic-SBP (per 1 mmHg)	0.000 (0.000–0.000)	−0.078	0.036
Current smoker (no = 0, yes = 1)	−0.002 (−0.004–0.001)	−0.160	<0.001
Diabetes mellitus (no = 0, yes = 1)	−0.005 (−0.008–0.001)	−0.114	0.006

Variables of simple regression analysis for Lg(eGFR) include age, gender (female = 0, male = 1), course, diabetes mellitus (no = 0, yes = 1), current smoker (no = 0, yes = 1), alcohol intake (no = 0, yes = 1), BMI, hemoglobin, albumin, calcium×phosphate, iPTH, uric acid, cholesterol, triglyceride, HDL-C, LDL-C, homocysteine, urinary sodium excretion, proteinuria and Clinic-SBP. Variables of simple regression analysis for LVMI, E/A, cIMT include age, gender (female = 0, male = 1), course, diabetes mellitus (no = 0, yes = 1), current smoker (no = 0, yes = 1), alcohol intake (no = 0, yes = 1), BMI, hemoglobin, albumin, calcium×phosphate, iPTH, uric acid, cholesterol, triglyceride, HDL-C, LDL-C, homocysteine, urinary sodium excretion, proteinuria, eGFR and Clinic-SBP. All variables with significant associations were included in the multivariable regression analysis. cIMT: Carotid intima-media thickness; DBP: Diastolic blood pressure; eGFR: Estimated glomerular filtration rate; iPTH: Intact parathyroid hormone; LVMI: Left ventricular mass index; SBP: Systolic blood pressure; peak velocities of early (E) and late (A) trans-mitral flow and deceleration time.

**Table 4:** Multivariable linear regression analysis for total homocysteine concentrations.

Variables	Unstandardized Coefficients B (95% Confidence Interval)	Standardized Coefficients Beta	*p* Value
Adjusted *R*^2^ = 0.397			
eGFR-MDRD (per 1 mL/min/1.73 m^2^)	−0.063 (−0.081–0.046)	−0.313	<0.001
Uric acid (per 1 mmol/L)	0.011 (0.006–0.016)	0.164	<0.001
Albumin (per 1 g/L)	0.234 (0.155–0.313)	0.194	<0.001
Gender (female = 0; male = 1)	3.467 (2.078–4.857)	0.164	<0.001
Hemoglobin (per 1 g/L)	−0.038 (−0.066–0.010)	−0.111	0.008
Calcium×Phosphate (mg^2^/dL^2^)	0.088 (0.017–0.159)	0.091	0.015

Variables of simple regression analysis for total homocysteine concentrations include age, gender (female = 0, male = 1), course, diabetes mellitus (no = 0, yes = 1), current smoker (no = 0, yes = 1), alcohol intake (no = 0, yes = 1), BMI, hemoglobin, albumin, calcium×phosphate, iPTH, uric acid, cholesterol, triglyceride, HDL-C, LDL-C, urinary sodium excretion, proteinuria, eGFR and Clinic-SBP. All variables with significant associations were included in multivariable regression analysis.

**Table 5:** Multivariable linear regression analysis for proteinuria.

Variables	Unstandardized Coefficients B (95% Confidence Interval)	Standardized Coefficients Beta	*p* Value
Dependent variable: Lg(proteinuria by MDRD formula) (g/L) (Adjusted *R*^2^ = 0.454)			
Age (per 1 year)	−0.007 (−0.011–0.003)	−0.165	0.001
Diabetes mellitus (no = 0, yes = 1)	0.181 (0.033–0.329)	0.017	0.017
Albumin (per 1 g/L)	−0.051 (−0.058–0.044)	−0.612	<0.001
Triglyceride (per 1 mmol/L)	0.068 (0.030–0.106)	0.149	<0.001
eGFR-MDRD (per 1 mL/min/1.73 m^2^)	−0.003 (−0.004–0.001)	−0.193	<0.001
Clinic-SBP (per 1 mmHg)	0.003 (0.000–0.005)	0.106	0.019

Variables of simple regression analysis for Lg(proteinuria by MDRD formula) include age, gender (female = 0, male = 1), course, diabetes mellitus (no = 0, yes = 1), current smoker (no = 0, yes = 1), alcohol intake (no = 0, yes = 1), BMI, hemoglobin, albumin, calcium×phosphate, iPTH, uric acid, cholesterol, triglyceride, HDL-C, LDL-C, homocysteine, urinary sodium excretion, proteinuria, eGFR and Clinic-SBP. All variables with significant associations were included in the multivariable regression analysis.
